# Populations of a Susceptible Amphibian Species Can Grow despite the Presence of a Pathogenic Chytrid Fungus

**DOI:** 10.1371/journal.pone.0034667

**Published:** 2012-04-05

**Authors:** Ursina Tobler, Adrian Borgula, Benedikt R. Schmidt

**Affiliations:** 1 Institute of Evolutionary Biology and Environmental Studies, University of Zurich, Zurich, Switzerland; 2 Büro für Naturschutzbiologie, Lucerne, Switzerland; 3 KARCH, Neuchâtel, Switzerland; Australian Wildlife Conservancy, Australia

## Abstract

Disease can be an important driver of host population dynamics and epizootics can cause severe host population declines. *Batrachochytrium dendrobatidis* (*Bd*), the pathogen causing amphibian chytridiomycosis, may occur epizootically or enzootically and can harm amphibian populations in many ways. While effects of *Bd* epizootics are well documented, the effects of enzootic *Bd* have rarely been described. We used a state-space model that accounts for observation error to test whether population trends of a species highly susceptible to *Bd*, the midwife toad *Alytes obstetricans*, are negatively affected by the enzootic presence of the pathogen. Unexpectedly, *Bd* had no negative effect on population growth rates from 2002–2008. This suggests that negative effects of disease on individuals do not necessarily translate into negative effects at the population level. Populations of amphibian species that are susceptible to the emerging disease chytridiomycosis can persist despite the enzootic presence of the pathogen under current environmental conditions.

## Introduction

Parasites and pathogens can be important drivers of host population dynamics by altering host behaviour, demography or genetics [1]–[4]. The most extreme effect of a pathogen on the host is host extinction [5]. However, host extinction is rare for two main reasons. First, parasites and their hosts generally share a common evolutionary history and extinction of either antagonist is hence unlikely [6]. Second, when host density declines, the pathogen's transmission rate is also expected to drop, unless there is frequency-dependent transmission or a reservoir host [5]. Emerging diseases, however, are different from established pathogens because pathogen interactions with novel hosts are unpredictable. In the most extreme –albeit rare– case they can lead to host population declines and extinctions [7], [8].

While the effects of disease on vital rates of captive populations are well documented, the effects of disease on dynamics of wild populations are still poorly understood [2], [4], [9], [10]. For example, a reduction in population size is only expected if pathogen-induced mortality is additive rather than compensatory [4], [11]. If pathogen-induced mortality is additive, then overall mortality is the sum of pathogen-induced mortality plus mortality inflicted by all other causes. In contrast, if mortality is compensatory, then increased mortality due to the presence of a pathogen is countered by a reduction in mortality due to other causes, often in a density-dependent manner [12]. Here, our goal is to contribute to a better understanding of host population dynamics influenced by an emerging infectious disease.

An emerging pathogen of amphibians, the chytrid fungus *Batrachochytrium dendrobatidis* (hereafter *Bd*), has contributed to amphibian declines and extinctions on most continents [13]–[15]. Many amphibian populations have collapsed after emergence of the pathogen, which is still appearing in new areas [16]–[18]. However, host extinction is not the only outcome of *Bd* emergence in new localities. The amphibian host-chytrid pathogen models by Briggs et al. [19], [20] suggest that enzootic *Bd*-infection may initially cause a reduction in abundance, but thereafter, populations remain stationary (i.e., mean abundance does not change anymore). Yet, due to a lack of time series data on the abundance of amphibian populations coexisting with enzootic *Bd*, we cannot state with certainty that amphibian populations with enzootic *Bd*-infection are stationary. In contrast to the model predictions, some mark-recapture studies have shown that enzootic *Bd* depresses both individual survival and population growth rates [21]–[23].

Our goal was to quantify the effects of enzootic *Bd* on populations of an amphibian species that is known to be susceptible to *Bd* [24]–[26]. Ideally, one would compare population sizes before and after the emergence of *Bd* in a population. Unfortunately, such data are rarely available [16], [17], [27]. We compare population monitoring data from sites where *Bd* is present with population monitoring data from sites where *Bd* was not detected. We analyse short time series of counts of calling males from 26 populations of the common midwife toad, *Alytes obstetricans*, in the Swiss canton Lucerne. Assuming that *Bd* leads to chytridiomycosis-induced mortality of individuals [24]–[26] and that mortality is additive rather than compensatory [4], [11], we expect to find declining populations in the presence of the pathogen while populations free of the pathogen should be either stationary or growing. The pathogen has been present in Switzerland since at least the early 1980 s [28], is widespread, and prevalence often high ([29], U. Tobler & B. R. Schmidt, unpublished data). Although no chytridiomycosis-induced mass mortality has been observed in Switzerland, including our study area, we know that *Bd*-associated mortality occurs in the field because we have detected dead metamorphs at our study sites that tested positive for *Bd* (U. Tobler, C. C. Geiger & B. R. Schmidt, unpublished data). Additionally, high (up to 90%) *Bd*-related mortality has been reported in a laboratory experiment on postmetamorphic *Alytes obstetricans* [25]. Although mortality in the field may be lower than in the laboratory, these results led us to expect *Bd*-induced population declines.

*Alytes obstetricans* is listed as “endangered” (IUCN category EN) on the Swiss national red list of threatened amphibians [30]. The species is therefore the target of conservation action [31]. Knowing whether and to what degree *Bd* poses a threat to *Alytes* population survival in this area is vital in order to develop suitable conservation strategies. Currently, no habitat mitigation methods are available barring ex-situ captive breeding although mitigation methods using antifungal chemicals or bacterial treatments are currently being tested [32]. With this study, we aim to test whether or not chytridiomycosis represents an additional threat to host populations of an endangered species.

## Materials and Methods

### Study sites

**Figure 1:**
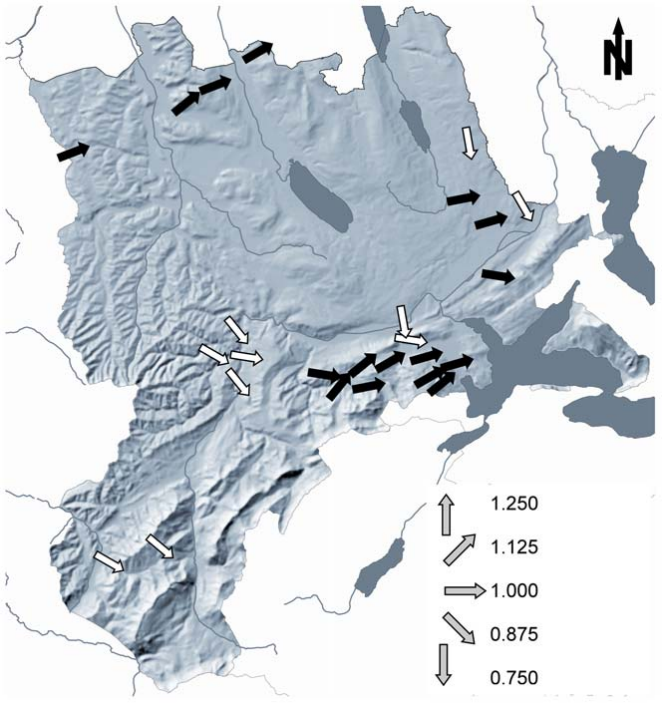
Topographic map of the canton Lucerne and study sites. Lakes are shaded in grey. White arrows mark sites where we did not detect *Bd*; black arrows mark *Bd*-positive sites. The slope of the arrows reflect population growth rates (Table 2).

Based on the availability of population count data, 26 sites in canton Lucerne, Switzerland, were included in the analysis. All sites are situated between 46.871° and 47.258° N and 7.882° and 8.382° E (Figure 1) and along an altitudinal gradient ranging from 402 to 1330 m.a.s.l. Mean summer temperatures range from 12.3 to 17.5°C [33]. Habitat types include quarries, ponds in open meadows, garden ponds, fire water reservoirs, and alpine and pre-alpine streams.

### Population monitoring

Populations of *Alytes obstetricans* were regularly monitored as part of the midwife toad action plan of the Swiss canton Lucerne [34], [35]. Counts of the number of calling *Alytes obstetricans* males were obtained every year from 2002 to 2008 except for four sites where *Alytes obstetricans* was reintroduced and calling males only occurred after 2002. Every site was visited at least twice a year by experienced volunteers. Volunteers were free to choose the nights for the monitoring, but advised to do so during optimal warm and humid nights when detectability of *Alytes obstetricans* is high because many males are calling [36]. The number of calling males, an index to population size [37], was recorded during every visit. We used the highest number of calling males within a year in the analysis. In our analysis of population trends, detectability may vary among years (the statistical model accounts for observation error; see below) but we assume that detectability shows no temporal trend such that population trends can be reliably estimated from counts of calling males [38], [39]. Trend estimates are unlikely to depend on *Bd* infection status because observers were unaware of *Bd* infection status of a site. Hence, we can exclude an interaction between *Bd* presence and detectability that would have biased our conclusion on the impact of *Bd* on population trends. In addition to the data on the numbers of calling males, presence or absence of tadpoles (based on visual encounter surveys [40]) was noted; this provided us with the explanatory variable “number of years tadpoles were observed”, which indicates the number of years in which tadpoles were observed in the pond.

### Sampling for Bd

During summer 2007 and spring 2008 or 2009, all sites were sampled for the presence of *Bd* in the amphibian population. To test the populations for *Bd*, 16 to 47 (mean ± SD: 26.0±6.6) amphibians were caught and swabbed with a sterile rayon swab (Copan Italia S.p.A., Brescia, Italy). Exceptions are sites “Sagerhüsli”, where only two dead *Alytes* metamorphs were sampled, and “Hombrig”, where sample size was six. Because both these sites tested positive for *Bd* we do not expect the small sample sizes to affect our results because the goal was to determine presence or absence of *Bd*. Sample size at sites where we did not detect *Bd* was 26.5±7.6 (mean ± SD). Hence, we cannot exclude that we may have missed *Bd* at some sites where prevalence was low (with a sample size of *n* = 26 a prevalence of 10% may not be detected; see [41]). Sampling was done opportunistically, i.e. all available amphibian species that could be captured were tested for *Bd*. Apart from midwife toads (where we swabbed tadpoles), the other amphibians sampled (always adults) were the fire-bellied toad *Bombina variegata*, the waterfrogs *Pelophylax lessonae* and *Pelophylax esculentus* (the two taxa were pooled because they form a hybridogenetic complex [42]), the alpine newt *Mesotriton alpestris* and the palmate newt *Lissotriton helveticus* (scientific names are based on [43]). To swab tadpoles, we made five swipes across the mouthparts. To sample adult amphibians, we swiped the underside of each foot five times and the ventral abdominal skin five times for a total of 25 swipes per amphibian. In 6 out of 11 sites where we did not detect *Bd*, samples were exclusively obtained from *Alytes* tadpoles. Due to the prolonged larval period *Alytes* tadpoles are more likely to be infected than any other species or life stage in this system (see results). Standard hygiene recommendations were followed during field work [44], [45].

We followed the rt-PCR protocol of Boyle et al [46] for the extraction and analysis of *Bd*-DNA from swabs. We used *Bd*-specific primers and standards to quantify the amount of *Bd*-DNA (infection load). To prevent inhibition by the extraction reagent, the extractions were diluted 1∶10 with water prior to PCR analysis. Hence, we calculated the original zoospore equivalent by multiplying the PCR output by 10. We ran each sample twice and the PCR was repeated if the two wells returned unequal results. Reactions below 0.1 genomic equivalents were scored *Bd*-negative [25].

### Statistical analysis

We tested for differences in prevalence of *Bd* among the sampled species using a generalised linear mixed model (GLMM) with a binomial error distribution. We tested for differences in infection intensity among species using a linear mixed model (LMM) with a normal error distribution. In both analyses, we used site as grouping (random) variable. Both analyses were done in R 2.8.1 [47].

We used WinBUGS 1.4 [48] to fit a a state-space model to the 26 time series [49], [50] and to assess whether the presence of *Bd* and the number of years tadpoles were observed affected population trends. State-space models disentangle the effects of the biological process and the observation process and thereby account for observation error. Modeling followed closely the approach described in Kéry & Schaub [50]. We built a model that estimates the observation and biological process at two hierarchical levels. The time series counts c_i,t_ are described by
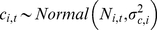
(1)where N_i,t_ is the unobserved true population size of site *i* at time *t* and 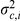 the observation variance at site *i*. This part of the model describes the observation process and removes observation error from the time series. The biological process is described by
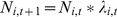
(2)whereλ_i,t_ is the population growth rate of site *i* at time *t* which is assumed to be have a normal distribution
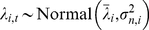
(3)where 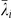 is the mean population growth rate at site *i* and 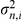 is the process variance at site *i*. 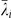 was further modeled as a function of site-specific presence of *Bd* (Bd_i_) and of the site-specific number of years tadpoles were observed (tad_i_) using a linear relationship:
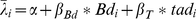
(4)


Uniform priors were used for the observation (U(0, 30) for each site) and for the process standard deviations (U(0, 10) for each site). A normal prior with a wide variance N(0, 100) was used for α, while for both β we used a uniform prior U(−5,5). Further, we used Normal priors for the population size at the first occasion for each site with variance 1 and the mean equal to the site-specific counts in that year. We ran three independent MCMC chains for 25,000 iterations, each with a burn-in of 10,000 iterations. The chains were thinned by a thinning factor of three. Convergence was assumed if the Gelman-Rubin statistic Rhat<1.1 [51].

### Ethics statement

The experiment was conducted under permit number 110/2007 by the veterinary office of the canton Zurich; collecting permits were provided by the office for Landwirtschaft und Wald (lawa) of the canton Lucerne.

## Results

We detected *Bd* in 16 out of the 26 (61.5%) sites and in 16.5% of all sampled amphibians, including the sites where we did not detect *Bd* (Table 1). In sites where *Bd* was detected, 28% of all sampled amphibians tested positive for *Bd*. *Bd* was not found in any site in the Entlebuch valley, which encompassed the south-western cluster of populations (Figure 1). The two dead metamorphs collected at Sagerhüsli in 2007 tested positive for *Bd* with infection intensities of 226.1 and 161.9 genomic equivalents. Four of five dead metamorphs collected at Schauensee in 2010 tested positive for *Bd* with an average infection intensity of 2.9±1.8 genomic equivalents (U. Tobler, C. C. Geiger & B. R. Schmidt, unpublished data). *Bd* data will be deposited at http://www.bd-maps.eu/.

**Table 1:** Estimates of observed infection prevalence and intensity in *Alytes* tadpoles and in all other species (pooled) for all study ponds.

	*Alytes*			other species		
Population	N	observed prevalence	infection intensity	N	observed prevalence	infection intensity
Ämmenmatt	7	0		23	0	
Ballwil	0	-	-	34	0.147	0.35 (±0.25)
Chalchloch	24	0		0	-	-
Chräuel	14	0.857	6.86 (±7.24)	17	0.294	2.39 (±3.16)
Egghütten	22	0	-	0	-	-
Ehrendingen	24	0	-	0	-	-
Einsamkeit	0	-	-	27	0.407	8.68 (±10.22)
Fontanne	21	0	-	0	-	-
Grisigen	20	1.000	2446.76 (±2764.18)	6	0.167	122.60
Hergiswald	17	0.706	21.58 (±26.01)	13	0.462	1.43 (±1.41)
Hilferenmättili	33	0	-	14	0	-
Hiltbrunnen	4	0.750	175.53 (±37.89)	23	0.652	16.99 (±26.07)
Hinter Rohren	6	0.333	215.96 (±58.52)	19	0	-
Hochrüti	0	-	-	16	0	-
Hohenrain	0	-	-	30	0	-
Hombrig	5	0.200	29.20	1	0	-
Lätten	1	0	-	33	0.121	1.86 (±1.95)
Linden	0	-	-	25	0.080	4.16 (±0.32)
Ottigenbüel	0	-	-	26	0.731	18.23 (±27.39)
Pfaffwil	0	-	-	24	0	-
Räschenhus	6	0.833	66.59 (±80.18)	19	0.211	1.11 (±0.60)
Rossei	25	0	-	0	-	-
Sagerhüsli	2	1.000	194.05 (±45.39)	0	-	-
Schauensee	0	-	-	30	0.233	3.44 (±4.21)
Schlagweiher	5	0.800	6032.2 (±3609.54)	20	0.500	235.36 ±(645.43)
Unter Utigen	0	1.000	-	27	0.296	42.97 (±85.57)

Prevalence is the proportion of infected individuals, infection intensity are mean zoospore equivalents (genomic equivalents) among infected individuals ±1 SD.

Within positive sites, *Alytes* tadpoles had higher observed infection prevalence than the other species (mean proportion of individuals carrying *Bd* per pond (mean, [range]): *A. obstetricans* tadpoles: 0.57 [0–0.95]; *Pelophylax* spp. adults: 0.27 [0.18–0.68]; *M. alpestris* adults: 0.24 [0–0.67]; *L. helveticus* adults: 0.14 [0–0.60]). Among infected individuals, infection intensity was higher in *Alytes* tadpoles than in the other species (*A. obstetricans:* 1266.5±322.2 (SE) genomic equivalents, *Pelophylax* spp. adults.: 29.1±11.4, *M. alpestris* adults: 46.1±36.2, *L. helveticus* adults: 17.9±9.3). The species differed significantly in observed infection prevalence (GLMM, z = −4.250, p = 0.0175) and intensity ( = *Bd* genomic equivalents; LMM, t = −3.551, p<0.001), respectively (Table 1).

*Alytes* populations had an average of 10.8±9.1 (SD) calling males (range within single years: 0–75 calling males; range of mean number of calling males per population: 2–43; Figure 2). Between 2002 and 2009, tadpoles were observed in 22 out of the 26 ponds during at least one visit; in those ponds where tadpoles were observed, they were seen in 4.8±1.7 years on average out of the 8 study years.

**Figure 2:**
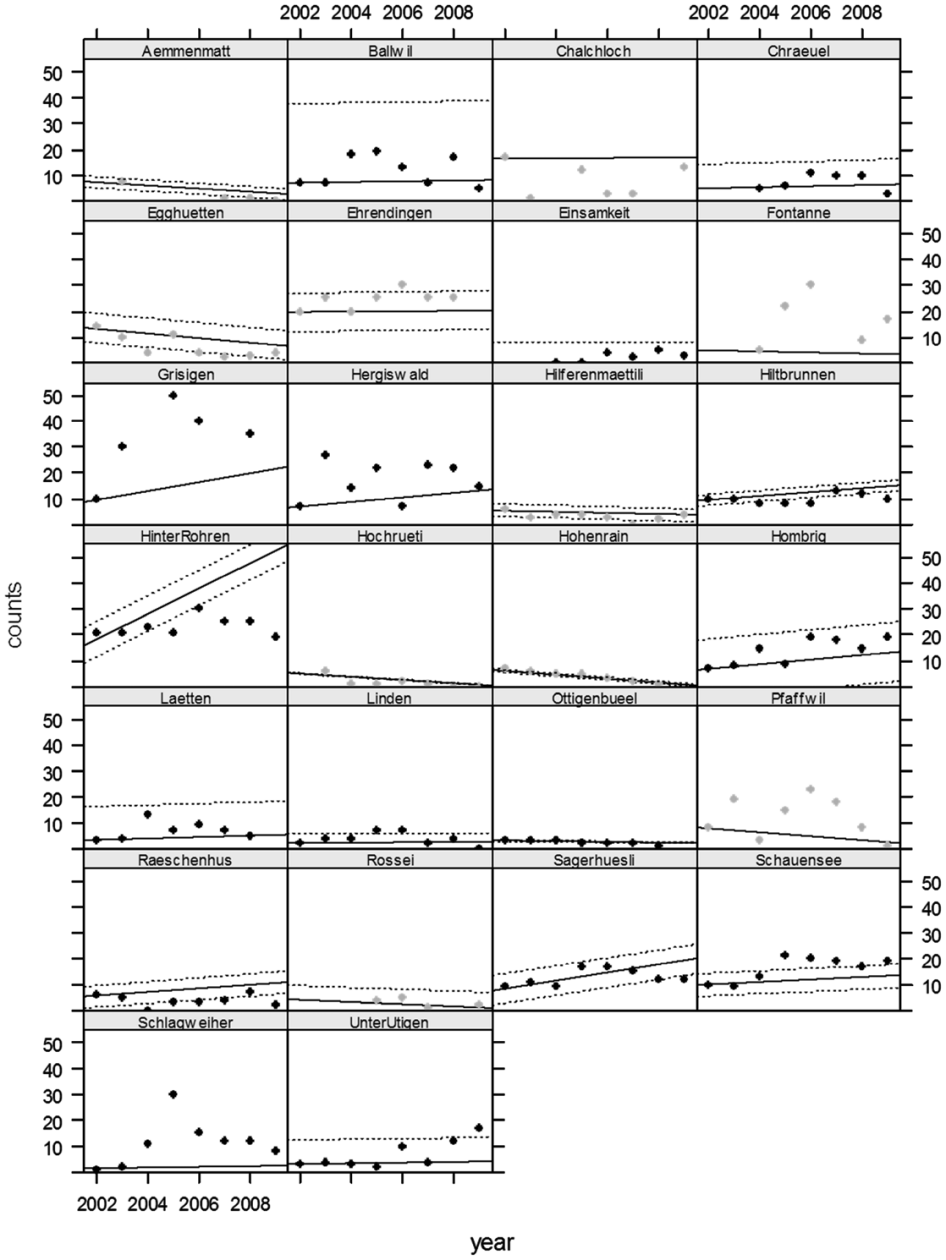
Observed counts and model-estimated trends per population and year. The circles show the observed counts of calling males, with sites where we did not detect *Bd* in grey and *Bd*-positive sites in black. The solid line shows the predicted population trend (with 95% CRI (dotted lines)).

Among populations, the mean population growth rates (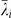; see equation 3) varied from 0.773 to 1.135; the average population growth rate of all populations during the 8-year study period was (mean ± SD) 0.987±0.105 (Table 2). This means that on average the number of calling males in the region was stationary. For 14 out of the 26 study sites, the 95% credible interval (CRI) for the estimated population trend included 1, i.e., the populations were neither growing nor declining (Figure 2). Populations that tested *Bd*-positive had on average a higher 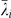
value than populations where we did not detect *Bd*. The difference in 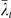 values was 0.191 (95% CRI 0.046–0.353; this is the regression coefficient for the effect of *Bd* on population growth rate (see equation 4 and Table 3)). Populations where tadpoles were observed in a higher number of study years had an increased growth rate compared to populations where tadpoles were not or only rarely observed; every year in which tadpoles were observed increased the growth rate by 0.024. The 95% CRI marginally included zero (95% CRI −0.007–0.051; Table 3). Repeating the analyses excluding the reintroduced populations did not alter the results qualitatively because parameter estimates were almost equal (results not shown).

**Table 2:** Population growth rates as predicted by the state-space model.

Population	*Bd* status	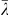	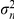	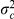
Aemmenmatt	0	0.871±0.063 (0.740–0.989)	5.271±12.056 (0.122–43.832)	3.140±21.863 (0.001–18.802)
Ballwil	1	1.013±0.052 (0.921–1.123)	0.536±1.391 (0.002–2.828)	43.395±57.075 (0.153–190.000)
Chalchloch	0	0.968±0.094 (0.757–1.128)	4.654±11.069 (0.008–38.782)	68.574±91.777 (1.212–327.005)
Chräuel	1	1.037±0.045 (0.953–1.132)	0.592±1.802 (0.002–3.098)	13.555±33.003 (0.007–83.283)
Egghütten	0	0.895±0.067 (0.752–1.017)	0.462±1.054 (0.002–2.515)	8.220±10.648 (0.014–35.720)
Ehrendingen	0	0.968±0.094 (0.757–1.128)	0.092±0.246 (0.001–0.446)	10.466±33.411 (0.003–58.931)
Einsamkeit	1	0.964±0.074 (0.843–1.121)	7.355±13.905 (0.005–52.242)	11.565±18.070 (0.491–52.482)
Fontanne	0	0.920±0.074 (0.756–1.049)	7.130±12.229 (0.008–45.040)	190.983±214.982 (0.056–761.725)
Grisigen	1	1.110±0.052 (1.015–1.212)	1.657±2.398 (0.107–6.936)	146.905±225.389 (0.032–791.812)
Hergiswald	1	1.086±0.045 (1.001–1.174)	0.518±1.668 (0.000–3.637)	140.024±120.709 (13.609–493.800)
Hilferenmättili	0	0.920±0.074 (0.756–1.049)	0.321±1.214 (0.000–1.946)	3.417±3.903 (0.437–12.890)
Hiltbrunnen	1	1.061±0.043 (0.980–1.144)	0.072±0.107 (0.000–0.308)	3.015±6.509 (0.002–13.750)
Hinter Rohren	1	1.135±0.062 (1.021–1.256)	0.098±0.121 (0.005–0.371)	9.704±23.339 (0.003–58.094)
Hochrüti	0	0.773±0.079 (0.610–0.936)	1.137±1.770 (0.209–4.371)	0.030±0.417 (0.000–0.248)
Hohenrain	0	0.773±0.079 (0.610–0.936)	0.061±0.114 (0.000–0.288)	0.501±1.150 (0.000–2.653)
Hombrig	1	1.086±0.045 (1.001–1.174)	0.166±0.323 (0.000–0.901)	16.112±22.979 (0.084–70.225)
Lätten	1	1.061±0.043 (0.980–1.144)	1.006±2.917 (0.001–5.459)	18.746±31.239 (0.035–90.531)
Linden	1	1.013±0.052 (0.921–1.123)	0.745±1.570 (0.004–3.461)	4.973±8.869 (0.006–25.180)
Ottigenbüel	1	0.964±0.074 (0.843–1.121)	0.098±0.356 (0.000–0.464)	0.297±0.716 (0.001–1.614)
Pfaffwil	0	0.846±0.062 (0.724–0.966)	1.044±2.237 (0.005–5.817)	91.589±107.273 (1.827–405.707)
Räschenhus	1	1.086±0.045 (1.001–1.174)	0.766±2.305 (0.019–4.071)	6.120±9.027 (0.456–26.250)
Rossei	0	0.846±0.062 (0.724–0.966)	3.107±8.811 (0.001–26.770)	8.242±31.220 (0.006–54.762)
Sagerhüsli	1	1.110±0.052 (1.015–1.212)	0.114±0.197 (0.001–0.488)	8.125±14.127 (0.023–38.550)
Schauensee	1	1.037±0.045 (0.953–1.132)	0.114±0.198 (0.004–0.464)	6.438±14.275 (0.002–37.001)
Schlagweiher	1	1.086±0.045 (1.001–1.174)	4.180±6.715 (0.030–21.820)	76.575±130.830 (0.024–457.207)
Unter Utigen	1	1.037±0.045 (0.953–1.132)	1.731±3.615 (0.010–10.011)	13.619±24.959 (0.003–71.705)

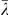
 is the average population growth rate. 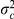 is the observation variance and 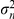 is the process variance. All estimates are given as means ± standard deviation with the 95% CRI in brackets. Bd status 0 means that Bd was not detected while Bd status 1 means that Bd was detected.

**Table 3:** Parameter estimates (equation 4), standard deviations and 95% CRI for the effects of the presence of *Bd* and the number of years in which tadpoles were observed on population growth rates.

parameter	mean	SD	95% CRI
α	0.773	0.079	0.610–0.936
*β_Bd_*	0.191	0.076	0.046–0.353
β_T_	0.024	0.015	−0.007–0.051

## Discussion

We found that *Alytes* populations in the Swiss canton Lucerne remained stable despite the presence of the pathogen, but that they were small with an average of only 10 calling males. *Bd*-positive populations did not have lower population growth rates than populations where we did not detect *Bd*. The result is robust even if we failed to detect *Bd* infection when it was present in some populations. If all populations where we did not detect *Bd* were *Bd*-positive, then the presence of *Bd* would still not lead to negative population trends since the average population growth rate is close to one.

The result was unexpected. The absence of negative effects of *Bd* on population trends contrasts strongly with many studies on other species that report dramatic negative effects of *Bd* on amphibian populations, including global extinction of species ([14]–[18], [22], but see [52]). It is even more surprising since *Alytes obstetricans* is known to be highly susceptible to *Bd* [23]–[25]. In a laboratory experiment, we showed that *Bd*-associated mortality of *Alytes obstetricans* shortly after metamorphosis was up to 90% [25]. Thus, there can be strong individual-level effects of *Bd*.

Population models suggest that high juvenile mortality lowers population growth rates in species with complex life cycles [53]–[56]. Hence, we expected that high chytridiomycosis-associated juvenile mortality in *Alytes obstetricans* in the laboratory [25] would lead to population declines. Even though we observed *Bd*-infected dead metamorphs in the field, there was apparently no effect of *Bd*-associated juvenile mortality on population trends. There are several possible explanations why there were no population-level effects of *Bd* in the field despite the strong individual-level effects in the laboratory [25]. The explanations are not mutually exclusive.

The first explanation is based on the fact that environmental conditions, especially those related to altitude and temperature, may mediate the effects of *Bd* on amphibian populations [26], [57], [58]. Under the prevailing environmental conditions, juvenile mortality in the field may be lower than in the laboratory [25]. It may be that environmental conditions, especially climate, in our study area may be such that there is some *Bd*–induced mortality of individuals but no population declines. However, we do not think that the populations that we studied experienced environmental conditions hostile to *Bd*. First, some dead and *Bd*-positive metamorphs were observed at two of our study sites. Admittedly, metamorphs found dead in the field had very low *Bd* loads perhaps indicating a cause of death other than chytridiomycosis (we did not do post mortem examinations to determine the cause of death). Second, one fifth of our populations were within the summer temperature range within which fatal chytridiomycosis is observed in Spain [26]. Further, because *Bd* occurred at all elevations and thus all climate regimes within our study region, we can exclude altitude as being confounded with *Bd* presence.

A second explanation may be that *Bd*-induced mortality could be compensatory rather than additive when *Bd* is enzootic. A decline in population size is only expected if mortality due to disease is additive, i.e. individuals die that would not die for other reasons in the absence of the disease [11]. While disease-induced mortality during *Bd* epizootics is obviously additive (e.g. when *Bd* epidemics caused *Alytes obstetricans* population declines in Spain [24]), this may not be the case for enzootic *Bd*. Compensatory mortality can result in a lack of disease effects on host abundance [4], [11]. If mortality is compensatory, increased mortality due to the presence of *Bd* would have to be countered by a reduction in mortality due to other causes, often in a density-dependent manner [12]. Density-dependence in the terrestrial stages of amphibian populations may indeed occur [59]–[61].

The third explanation may be that effects of *Bd* on abundance occurred in the past and are no longer measurable. The Briggs et al. host-pathogen models [19], [20] suggests that amphibian populations may decline to lower abundance after the emergence of *Bd* but remain stationary at a smaller size after *Bd* became enzootic. Indeed, strong *Alytes obstetricans* population declines were observed in our study area in the 1980 s and 1990 s [31]. Since *Bd* has occurred in Switzerland since at least the early 1980 s [28], it may be that *Bd* contributed to these declines in the past. Today, the *Alytes obstetricans* populations may have reached the stationary state predicted by the models such that an effect of *Bd* on abundance is no longer detectable. This is also supported by the low infection intensities observed in the field today. If populations have stabilized at lower abundance – which is highly plausible given the small population sizes observed in this study – they may now be less resilient [62] or more prone to environmental and/or demographic stochasticity because of reduced abundance. It is also possible that unusual environmental conditions could interact with *Bd* to affect populations in the future.

Although there are several possible explanations for why we did not observe the expected negative effect of *Bd* on abundance, the question remains open as to why *Bd*-positive populations had higher growth rates than *Bd*-negative populations. The more years tadpoles were observed at a given site, the more likely the population was to grow (Table 2). This may suggest that recruitment may determine population growth [55], [63]. Hence, the compensatory mechanism that we alluded to above may simply be increased recruitment. Increased recruitment of *Bd*-positive populations of *Anaxyrus* (*Bufo*) *boreas* may allow these populations to persist despite the presence of *Bd* [22].

In conclusion, we demonstrated that populations of a species that is susceptible to an emerging pathogen can grow despite a high prevalence of the pathogen. Evidently, individual-level effects of disease (mortality of individuals) did not translate into population-level effects (negative population growth rates). Our results are phenomenological and we do not know the mechanisms that allow the populations to persist. Understanding why an amphibian species that is known to be susceptible to *Bd* can have growing populations despite high prevalence of the pathogen would be a key to successful mitigation of the effects of chytridiomycosis.

## References

[1] Anderson RM, May RM (1979). Population biology of infectious diseases: Part I.. Nature.

[2] Tompkins DM, Begon M (1999). Parasites can regulate wildlife populations.. Parasitol Today.

[3] Ibelings BW, De Bruin A, Kagami M, Rijkeboer M, Brehm M (2004). Host parasite interactions between freshwater phytoplankton and chytrid fungi (Chytridiomycota).. J Phycol.

[4] Jolles AE, Etienne RS, Olff H (2006). Independent and competing disease risks: Implications for host populations in variable environments.. Amer Nat.

[5] de Castro F, Bolker B (2005). Mechanisms of disease-induced extinction.. Ecol Lett.

[6] Ebert D, Hamilton WD (1996). Sex against virulence: the coevolution of parasitic diseases.. Trends Ecol Evol.

[7] Ebert D (1994). Virulence and local adaptation of a horizontally transmitted parasite.. Science.

[8] Read AF (1994). The evolution of virulence.. Trends Microbiol.

[9] Tompkins DM, Dobson AP, Arneberg P, Begon ME, Cattadori IM, Hudson PJ, Rizzoli A, Grenfell BT, Heesterbeek H, Dobson AP (2001). Parasites and host population dynamics.. The ecology of wildlife diseases.

[10] Deem SL, Ezenwa VO, Ward JR, Wilcox BA, Ostfeld RS, Keesing F, Eviner VT (2008). Research frontiers in ecological systems: evaluating the impacts of disease on ecosystems.. Infectious disease ecology: effects of ecosystems on disease and of disease on ecosystems.

[11] Burnham KP, Anderson DR (1984). Tests of compensatory vs. additive hypotheses of mortality in mallards.. Ecology.

[12] Lebreton JD (2005). Dynamical and statistical models for exploited populations.. Austral New Zealand J Stat.

[13] Stuart SN, Chanson JS, Cox NA, Young BE, Rodrigues ASL (2004). Status and trends of amphibian declines and extinctions worldwide.. Science.

[14] Skerratt LF, Berger L, Speare R, Cashins S, McDonald KR (2007). Spread of chytridiomycosis has caused the rapid global decline and extinction of frogs.. EcoHealth.

[15] Fisher MC, Garner TWJ, Walker SF (2009). Global emergence of *Batrachochytrium dendrobatidis* and amphibian chytridiomycosis in space, time, and host.. Annu Rev Microbiol.

[16] Laurance WF, McDonald KR, Speare R (1996). Epidemic disease and the catastrophic decline of Australian rain forest frogs.. Conserv Biol.

[17] Lips KR, Brem F, Brenes R, Reeve JD, Alford RA (2006). Emerging infectious disease and the loss of biodiversity in a neotropical amphibian community.. Proc Nat Acad Sci USA.

[18] Vredenburg VT, Knapp RA, Tunstall TS, Briggs CJ (2010). Dynamics of an emerging disease drive large-scale amphibian population extinctions.. Proc Nat Acad Sci USA.

[19] Briggs CJ, Vredenburg VT, Knapp RA, Rachowicz LJ (2005). Investigating the population-level effects of chytridiomycosis: An emerging infectious disease of amphibians.. Ecology.

[20] Briggs CJ, Knapp RA, Vredenburg VT (2010). Enzootic and epizootic dynamics of the chytrid fungal pathogen of amphibians.. Proc Nat Acad Sci USA.

[21] Pilliod DS, Muths E, Scherer RD, Partelt PE, Corn PS (2010). Effects of amphibian chytrid fungus on individual survival probability in wild boreal toads.. Conserv Biol.

[22] Muths E, Scherer RD, Pilliod DS (2011). Compensatory effects of recruitment and survival when amphibian populations are perturbed by disease.. J Appl Ecol.

[23] Longo AV, Burrowes PA (2010). Persistence with chytridiomycosis does not assure survival of direct-developing frogs.. EcoHealth.

[24] Bosch J, Martínez-Solano I, García-París M (2001). Evidence of a chytrid fungus infection involved in the decline of the common midwife toad (*Alytes obstetricans*) in protected areas of central Spain.. Biol Conserv.

[25] Tobler U, Schmidt BR (2010). Within- and among-population variation in chytridiomycosis-induced mortality in the toad *Alytes obstetricans*.. PLoS ONE.

[26] Walker SF, Bosch J, Gomez V, Garner TWJ, Cunningham AA (2010). Factors driving pathogenicity vs. prevalence of amphibian panzootic chytridiomycosis in Iberia.. Ecol Lett.

[27] Teacher AGF, Cunningham AA, Garner TWJ (2010). Assessing the long-term impact of *Ranavirus* infection in wild common frog populations.. Anim Conserv.

[28] Peyer NF (2010). Historical evidence for the presence of the emerging amphibian pathogen *Batrachochytrium dendrobatidis* (Longcore et al. 1999) in Switzerland. M.Sc. thesis.

[29] Garner TWJ, Walker S, Bosch J, Hyatt AD, Cunningham AA (2005). Chytrid fungus in Europe.. Emerg Infect Dis.

[30] Schmidt BR, Zumbach S (2005). Rote Liste der gefährdeten Amphibien der Schweiz.

[31] Borgula A, Zumbach S (2003). Verbreitung und Gefährdung der Geburtshelferkröte (*Alytes obstetricans*) in der Schweiz.. Z Feldherpetol.

[32] Woodhams DC, Bosch J, Briggs CJ, Cashins S, Davis LR (2011). Mitigating amphibian disease: strategies to maintain wild populations and control chytridiomycosis.. Front Zool.

[33] Hijmans RJ, Cameron SE, Parra JL, Jones PG, Jarvis A (2005). Very high resolution interpolated climate surfaces for global land areas.. Int J Climatol.

[34] Amt für Natur- und Landschaftsschutz (2000). Artenhilfsprogramm Geburtshelferkröte 2000–2009.

[35] Borgula A, Zuberbühler N (2010). Artenhilfsprogramm Geburtshelferkröte 2010–2019.

[36] Schmidt BR (2005). Monitoring the distribution of pond-breeding amphibians when species are detected imperfectly.. Aquat Conserv.

[37] Schmidt BR (2004). Declining amphibian populations: The pitfalls of count data in the study of diversity, distributions, dynamics, and demography.. Herpetol J.

[38] Bart J, Droege S, Geissler P, Peterjohn B, Ralph CJ (2004). Density estimation in wildlife surveys.. Wildl Soc Bull.

[39] Kéry M, Schmidt BR (2008). Imperfect detection and its consequences for monitoring for conservation.. Comm Ecol.

[40] Skelly DK, Richardson JL, Dodd CK (2009). Larval sampling.. In Amphibian ecology and conservation: a handbook of techniques.

[41] DiGiacomo RF, Koepsell TD (1986). Sampling for detection of infection or disease in animal populations.. J Amer Vet Med Assoc.

[42] Schmidt BR (1993). Are hybridogenetic frogs cyclical parthenogens?. Trends Ecol Evol.

[43] Vences M (2007). The Amphibian Tree of Life: Ideologie, Chaos oder biologische Realität?. Z Feldherpet.

[44] Schmidt BR, Furrer S, Kwet A, Lötters S, Rödder D, Hachtel M, Schlüpmann M, Thiesmeier B, Weddeling K (2009a). Desinfektion als Maßnahme gegen die Verbreitung der Chytridiomykose bei Amphibien.. In Methoden der Feldherpetologie.

[45] Schmidt BR, Geiser C, Peyer N, Keller N, von Rütte M (2009b). Assessing whether disinfectants against the fungus *Batrachochytrium dendrobatidis* have negative effects on tadpoles and zooplankton.. Amphibia-Reptilia.

[46] Boyle DG, Boyle DB, Olsen V, Morgan JAT, Hyatt AD (2004). Rapid quantitative detection of chytridiomycosis (*Batrachochytrium dendrobatidis*) in amphibian samples using real-time Taqman PCR assay.. Dis Aquat Org.

[47] R Development Core Team (2008). R: A language and environment for statistical computing.. http://www.R-project.org.

[48] Lunn DJ, Thomas A, Best N, Spiegelhalter D (2000). WinBUGS – a Bayesian modelling framework: concepts, structure, and extensibility.. Stat Comp.

[49] de Valpine P, Hastings A (2002). Fitting population models incorporating process noise and observation error.. Ecol Monogr.

[50] Kéry M, Schaub M (2012). Bayesian Population Analysis using WinBUGS: A hierarchical perspective.

[51] Gelman A, Hill J (2007). Data analysis using regression and multilevel/hierarchical models.

[52] Retallick RWR, McCallum H, Speare R (2004). Endemic infection of the amphibian chytrid fungus in a frog community post-decline.. PLoS Biol.

[53] Conroy SDS, Brook BW (2003). Demographic sensitivity and persistence of the threatened white- and orange-bellied frogs of Western Australia.. Pop Ecol.

[54] Hels T, Nachman G (2002). Simulating viability of a spadefoot toad *Pelobates fuscus* metapopulation in a landscape fragmented by a road.. Ecography.

[55] Lampo M, De Leo GA (1998). The invasion ecology of the toad *Bufo marinus*: from South America to Australia.. Ecol Appl.

[56] Di Minin E, Griffiths RA (2011). Viability analysis of a threatened amphibian population: modelling the past, present and future.. Ecography.

[57] Bosch J, Carrascal LM, Duran L, Walker S, Fisher MC (2007). Climate change and outbreaks of amphibian chytridiomycosis in a montane area of Central Spain; is there a link?. Proc Roy Soc B.

[58] Puschendorf R, Hoskin CJ, Cashins SD, McDonald KR, Skerratt LF (2011). Environmental refuge from disease-driven amphibian extinction.. Conserv Biol.

[59] Altwegg R (2003). Multistage density dependence in an amphibian.. Oecologia.

[60] Berven KA (2009). Density dependence in the terrestrial stage of wood frogs: evidence from a 21-year population study.. Copeia.

[61] Patrick DA, Harper EB, Hunter ML, Calhoun AJK (2008). Terrestrial habitat selection and strong density-dependent mortality in recently metamorphosed amphibians.. Ecology.

[62] Jolles AE, Cooper DV, Levin SA (2005). Hidden effects of chronic tuberculosis in African buffalo.. Ecology.

[63] Grafe TU, Kaminsky SK, Bitz JH, Lussow H, Linsenmair KE (2004). Demographic dynamics of the afro-tropical pig-nosed frog, *Hemisus marmoratus*: effects of climate and predation on survival and recruitment.. Oecologia.

